# Prospective randomized trial of triple port laparoscopic cholecystectomy combined with choledochoscopic common bile duct exploration and primary closure for acute abdominal pain

**DOI:** 10.1038/s41598-026-37034-8

**Published:** 2026-01-22

**Authors:** Xirang Wang, Jian Kang, Yuxiang Li, Xiaofeng Sun, Jun Zhang, Yunpeng Wu, Hehui Tao, Li Wang, Ruizhou Rong, Miao Wang, Kang Liu, Zhen Ban

**Affiliations:** 1 Department of General Surgery, Beijing Fengtai Youanmen Hospital, Beijing, 100069 China; 2 Department of General Surgery, Beijing Huimin Hospital, Beijing, 100054 China

**Keywords:** Choledocholithiasis, CBDS, Laparoscopy, Choledochoscopic exploration, T-tube drainage, Prospective study, Gastroenterology, Medical research

## Abstract

To evaluate the clinical efficacy of laparoscopic common bile duct exploration (LCBDE) combined with choledochoscopy and primary closure without T-tube drainage in managing acute abdominal pain caused by choledocholithiasis (common bile duct stones, CBDS) and cholecystolithiasis with acute cholecystitis. A single-center prospective study was conducted at the Department of General Surgery, Beijing Fengtai Youanmen Hospital, from April 2024 to February 2025. Sixty-one patients with acute abdominal pain due to CBDS and cholecystolithiasis with acute cholecystitis were randomized into two groups: T-tube-free group (n = 35), Triple-port laparoscopic cholecystectomy (LC) + LCBDE with primary closure; T-tube group (n = 26), Four-port LC + LCBDE with T-tube drainage. Perioperative outcomes were compared between the groups. Baseline characteristics were comparable (all *P* > 0.05). The T-tube-free group demonstrated superior outcomes in operative time, intraoperative blood loss, postoperative pain, duration of abdominal drainage, and hospital stay (all *P* < 0.05). Each group had one case of biliary leakage, both resolved conservatively. No mortality, pancreatitis, conversion to open surgery, residual stones, biliary hemorrhage, or strictures occurred in either group. Triple-port LC combined with LCBDE and primary closure without T-tube drainage is safe and feasible for acute abdominal pain. Compared to T-tube drainage, this approach better aligns with the principles of enhanced recovery after surgery (ERAS).

## Introduction

The prevalence of cholecystolithiasis in China has demonstrated a consistent annual increase, with epidemiological data indicating that approximately 20% of adults harbor asymptomatic gallstones^1^. Notably, 85% of CBDS are secondary to gallbladder stones, where stone migration via gallbladder contraction may precipitate acute biliary obstruction. This pathological process can trigger severe complications including acute cholangitis (17.3%), acute pancreatitis (9.6%), and septic shock (3.1%)^2,3^. The surgical management of acute abdominal pain caused by CBDS and cholecystolithiasis with acute cholecystitis has evolved from traditional open procedures to minimally invasive techniques. Within this paradigm shift, the central surgical challenge has become the development of innovative approaches that minimize operative trauma while optimizing postoperative recovery^4,5^. Under the guidance of ERAS principles, our center has observed that while conventional four-port LCBDE with T-tube drainage remains prevalent for acute presentations, the triple-port LCBDE with primary closure remains underutilized in emergency settings. Through strategic optimization of port placement and refined common bile duct (CBD) incision/closure techniques, we have achieved reduced iatrogenic trauma and accelerated postoperative recovery. To rigorously evaluate the safety and feasibility of this approach, we conducted a prospective randomized controlled trial (RCT), the results of which are presented herein.

## Materials and methods

### General data

This trial was registered at China’s national registry for medical research (Chinese Medical Research Registration and Filing Information System, CMRRFIS), a primary registry in the WHO Registry Network (Registration number: MR-11-24-008651; Date of first registration: 06/03/2024). This prospective randomized controlled study was approved by the Ethics Committee of Beijing Fengtai Youanmen Hospital (Grant No. LL-2024-04) and conducted in accordance with the Helsinki Declaration. Patients or their families signed informed consent forms. Supported by the hospital’s research fund (Grant No. KY-2024-04), the study enrolled 61 patients with acute abdomen due to CBDS and cholecystolithiasis with acute cholecystitis from April 2024 to February 2025. The inclusion criteria were as follows: patients who were able to tolerate laparoscopic general anesthesia, had no history of previous laparotomy, and presented with acute symptom onset within one week. Conversely, exclusion criteria comprised patients deemed unfit for laparoscopic surgery, those with a prior laparotomy, or individuals with chronic or recurrent symptoms lasting for several months to years. Patients were randomly assigned to the T-tube-free group or the T-tube group using Research Randomizer (http://www.randomizer.org). Participants, care givers, and those assessing the outcomes were blinded to group assignment. The T-tube-free group (n = 35) underwent triple-port LCBDE with primary closure, versus the T-tube group (n = 26) managed with four-port LCBDE + T-tube placement. Baseline characteristics (gender, age, BMI, symptom duration, laboratory values [TBIL, DBIL, ALP, ALT, AST, AMY, WBC, ALB, CRP, PCT], CBD diameter, pain scores, comorbidities) showed no intergroup differences (all P > 0.05, Table 1).

**Table 1:** Preoperative general clinical data of the two groups.

	T-tube-free group(n = 35)	T-tube group (n = 26)	Statistic	*P*
Gender (number)			χ2 = 0.066	0.798
Male	20	14		
Female	15	12		
Age (years)	63.31 ± 17.02	62.42 ± 13.78	*t* = 0.219	0.827
BMI (Kg/m^2^)	24.74 ± 2.38	24.62 ± 3.16	*t* = 0.180	0.858
Symptom duration (days)	3.00(1.00,5.00)	3.00(2.00,5.00)	*z* = − 0.449	0.654
TBIL (μmol/L)	38.37 ± 18.02	45.77 ± 16.31	*t* = − 1.650	0.104
DBIL (μmol/L)	20.86 ± 10.51	24.31 ± 8.17	*t* = − 1.390	0.170
ALP (U/L)	205.51 ± 88.02	221.23 ± 108.72	*t* = − 0.624	0.535
ALT (U/L)	55.80 ± 26.46	54.85 ± 20.17	*t* = 0.154	0.879
AST (U/L)	61.97 ± 27.98	63.96 ± 29.37	*t* = − 0.269	0.789
AMY (U/L)	60.69 ± 31.15	59.31 ± 29.45	*t* = 0.175	0.862
WBC (10^9^/L)	11.63 ± 3.94	13.12 ± 3.33	*t* = − 1.555	0.125
ALB (g/L)	39.23 ± 4.63	37.5 ± 4.77	*t* = 1.424	0.160
CRP (mg/L)	39.91 ± 19.37	46.54 ± 19.81	*t* = − 1.308	0.196
PCT (ng/ml)	2.10 ± 0.92	2.32 ± 0.96	*t* = − 0.874	0.386
CBD diameter (mm)	11.99 ± 2.13	11.50 ± 2.39	*t* = 0.833	0.408
Preoperative pain scores (NRS)	4.51 ± 2.15	4.23 ± 1.36	*t* = 0.591	0.557
Comorbidities(number)			χ2 = 1.010	0.315
Yes	17	16		
No	18	10		

### Surgical procedure

All patients were preoperatively assessed by an anesthesiologist using the American Society of Anesthesiologists (ASA) score. The same surgical team performed all operations, with the chief surgeon positioned at the patient’s right head side and the assistant at the right tail side.

**Fig. 1:**
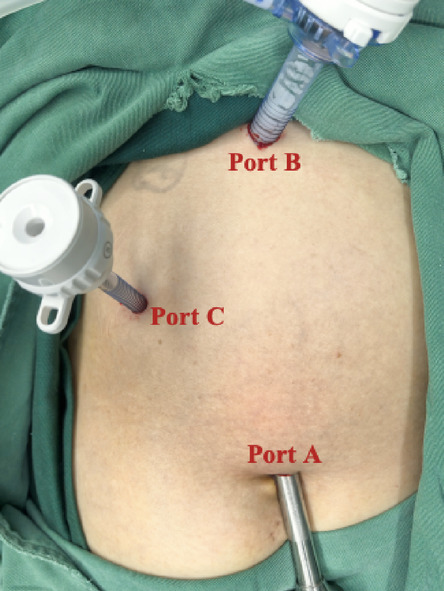
Standard port configuration for T-tube-free group.

**Fig. 2:**
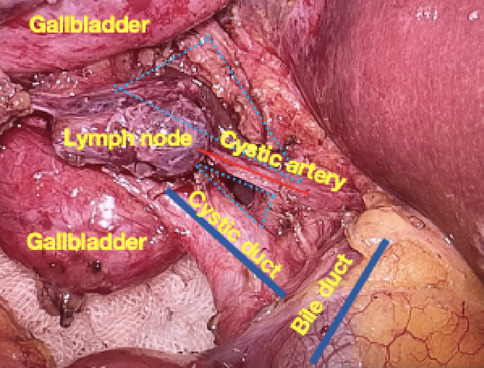
Intraoperative CVS criteria.

**Fig. 3:**
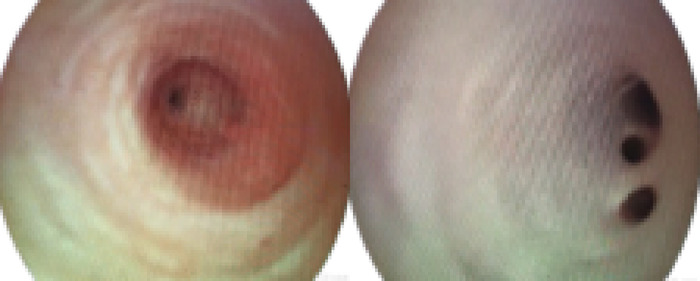
Choledochoscopic exploration of CBD.

**Fig. 4:**
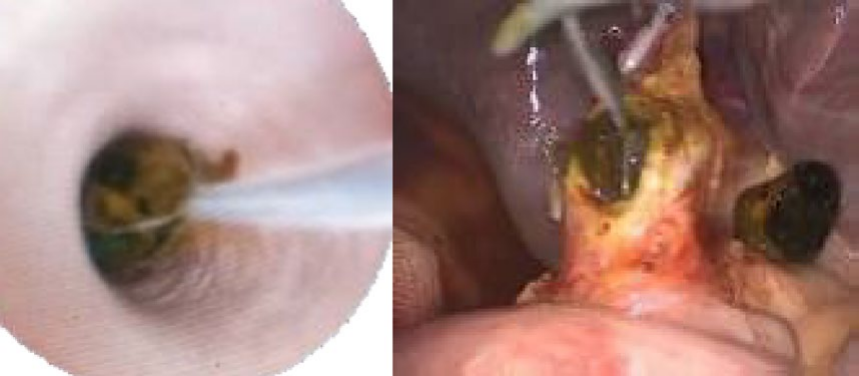
Stone retrieval under choledochoscopy.

**Fig. 5:**
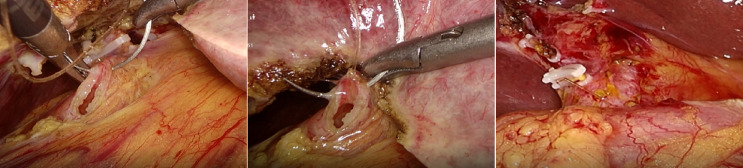
Primary closure of CBD with barbed suture.

In the T-tube-free group, a 10-mm umbilical incision (camera port, Port A) was created for CO_2_ pneumoperitoneum establishment and trocar insertion. Following diagnostic laparoscopy, two additional ports were placed under direct visualization: A 10-mm epigastric port (Port B) 1 cm below the xiphoid process; A 5-mm right subcostal port (Port C) 2 cm below the costal margin on the midclavicular line (Fig. 1). The gallbladder and hepatocystic triangle were carefully exposed. LC was performed adhering strictly to the Critical View of Safety (CVS) criteria (Fig. 2). The Calot’s triangle was dissected using a combination of sharp and blunt techniques until only the cystic duct and cystic artery were clearly identified, which were then clipped and divided. The gallbladder was dissected from its bed and placed in a disposable retrieval bag, temporarily positioned in the perihepatic space. The CBD was fully mobilized, and a longitudinal incision was made distal to the cystic duct–common hepatic duct confluence using an electrocautery hook in cut mode (power < 45 W). Bile efflux confirmed entry into the lumen. The incision was gently widened using laparoscopic dissectors. A choledochoscope was introduced (Fig. 3), revealing CBDS, which were extracted using a disposable stone retrieval basket (Fig. 4). Repeat choledochoscopy confirmed clearance of stones from the distal CBD (to the duodenal papilla) and proximal ducts (up to secondary hepatic branches), with no obstructions or masses detected (Fig. 3). The CBD incision was closed (Fig. 5) with a continuous 3-0 Polydioxanone (PDO) Barbed Absorbable Suture (stitch interval: 1.0–1.5 mm). A gauze compression test confirmed no bile leakage. The specimen was extracted via Port B. The abdominal cavity was irrigated with warm saline, and a closed-suction drain was placed in the subhepatic space via Port C.

**Fig. 6:**
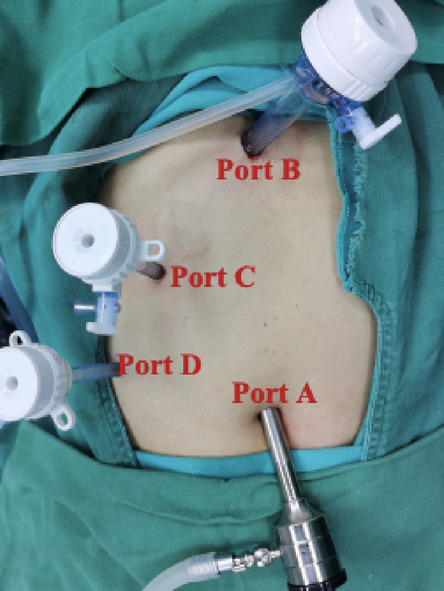
Standard port configuration for T-tube drainage group.

**Fig. 7:**
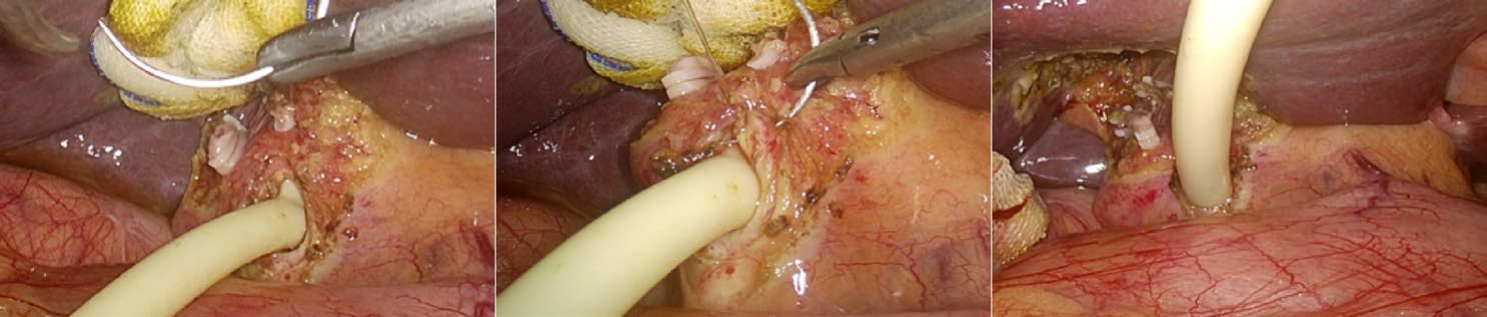
T-tube placement in the CBD.

In the T-Tube Drainage Group, port positions A (umbilical), B (subxiphoid), and C (right subcostal) mirrored those in the triple-port group, with the addition of port D: 5-mm trocar placed 2 cm below the right costal margin along the anterior axillary line (Fig. 6). The assistant utilized Port D to optimize retraction and exposure of Calot’s triangle. Following identical choledochoscopic stone extraction as the triple-port group, a T-tube was inserted into the CBD via Port C (Fig. 7). A closed-suction drain was positioned in the subhepatic space through Port D. The T-tube suture line was tested for watertight closure by instilling 10 mL of 0.9% sterile saline under direct visualization, with no evidence of extravasation.

### Outcome measures

The following parameters were prospectively recorded and compared between groups. Intraoperative outcomes: Operative time (minutes), Intraoperative blood loss (mL), CBD stone characteristics (number, maximum diameter in mm); Postoperative recovery: Pain scores (Visual Analog Scale,VAS 0-10), Time to first flatus (days), Duration of abdominal drainage (days), Length of hospital stay (days); Complications: Conversion to open surgery, Biliary hemorrhage, Bile leakage, Pancreatitis, Biliary stricture (diagnosed by MRCP at 3-month follow-up).

### Statistical analysis

Statistical analyses were performed using IBM SPSS Statistics 23.0 (IBM Corp., Armonk, NY, USA). Continuous variables were first assessed for normality using the Shapiro–Wilk test. For normally distributed data with homogeneous variance (evaluated by Levene’s test), intergroup comparisons were conducted using independent samples t-test. Non-normally distributed data were analyzed using the Mann–Whitney U test. Categorical variables were compared using the χ^2^ test or Fisher’s exact test, as appropriate. A two-tailed P-value < 0.05 was considered statistically significant.

## Results

The triple-port T-tube-free group demonstrated significantly better outcomes in operative time (shorter), intraoperative blood loss (less), postoperative day (POD) 1 pain scores (lower), duration of abdominal drainage (shorter), and hospital stay (reduced), with all differences being statistically significant (P < 0.05). However, there were no significant intergroup differences in the number of CBDS, maximum stone diameter, or time to first flatus (P > 0.05, Table 2). Each group had one case of bile leakage (Clavien–Dindo Grade I), which resolved spontaneously by POD3 (drainage fluid became clear and non-bilious), with drains successfully removed on POD4 in both cases. No instances of major intraoperative hemorrhage or conversion to open surgery occurred in either group. All patients had their abdominal drains removed prior to discharge. In the T-tube group, all tubes were removed 6–8 weeks postoperatively. Zero mortality in both cohorts. No cases of pancreatitis and biliary hemorrhage were observed. All patients underwent MRCP at 3 months postoperatively to evaluate for biliary strictures or residual stones. No such complications were observed.

**Table 2:** Comparison of intraoperative and postoperative indexes between the two groups.

	T-tube-free group(n = 35)	T-tube group (n = 26)	Statistic	*P*
Operative time(min)	88.94 ± 27.68	115.58 ± 32.83	*t* = − 3.432	0.001
Intraoperative blood loss (ml)	10.00(10.00,10.00)	10.00(10.00,20.00)	*z* = − 2.069	0.039
Number of CBDS (number)	4.00(3.00,6.00)	4.50(2.00,6.00)	*z* = − 0.037	0.971
Maximum diameter of CBDS (mm)	2.67 ± 0.86	2.98 ± 0.51	*t* = − 1.665	0.101
POD1 pain scores (VAS)	3.53 ± 0.64	3.97 ± 0.57	*t* = − 2.796	0.007
Time to first flatus (days)	1.15 ± 0.35	1.25 ± 0.30	*t* = − 1.105	0.274
Duration of abdominal drainage (days, ≤ 50 mL/day, non-bilious)	3.82 ± 0.42	4.69 ± 0.64	*t* = − 6.426	< 0.001
Length of hospital stay (days)	6.17 ± 0.47	6.82 ± 0.78	*t* = − 4.081	< 0.001

## Discussion

This prospective RCT represents a systematic comparison between triple-port T-tube-free LCBDE and conventional T-tube drainage in the management of acute abdominal pain secondary to CBDS and cholecystolithiasis with acute cholecystitis. Our findings demonstrate that the T-tube-free approach achieved statistically superior outcomes across multiple key parameters: shorter operative time (88.94 ± 27.68 vs. 115.58 ± 32.83 min, *P* = 0.001), reduced blood loss (median 10.00 [IQR 10.00–10.00] vs. 10.00 [10.00–20.00] mL, *P* = 0.039), lower POD1 surgical site pain scores (3.53 ± 0.64 vs. 3.97 ± 0.57, *P* = 0.007), earlier drain removal (3.82 ± 0.42 vs. 4.69 ± 0.64 days, *P* < 0.001), shorter hospitalization (6.17 ± 0.47 vs. 6.82 ± 0.78 days, *P* < 0.001). These results align with recent technical advancements in LCBDE^5–8^, particularly regarding trauma minimization through reduced-port strategies. The omission of T-tube placement and fixation shortened operative time by reducing suturing requirements. Although median intra-operative blood loss differed statistically between groups, the absolute values were identical (10 mL), indicating limited clinical relevance. Drain removal was governed by drainage volume, and the earlier attainment of the predefined output threshold in the study group translated into a shorter interval to drain extraction, which in turn contributed to the observed reduction in post-operative length of stay. Both groups received identical analgesia protocols: intravenous tramadol as needed. Both groups exhibited comparable complication rates, with one case each of self-limiting bile leakage (resolved by POD4). No instances of: major intraoperative hemorrhage, conversion to laparotomy; mortality, Pancreatitis, retained stones, biliary hemorrhage, or strictures within 3-month follow-up.

Endoscopic sphincterotomy (EST), while widely used, carries significant limitations: permanent disruption of sphincteric integrity, post-ERCP pancreatitis rates of 8.3–15.6%^9^, 2.3-fold greater stone recurrence versus surgical interventions^10^. Percutaneous transhepatic approaches remain niche due to: specialized equipment requirements and procedural complexity. Utilization in acute settings is limited to < 5% of cases^11^. In contrast, the one-stage LC and LCBDE, which combines gallbladder removal and common bile duct stone extraction, has become the mainstay of treatment. This approach preserves the integrity of the Oddi sphincter and achieves a CBDS clearance rate of over 95%^4,6^.

This study demonstrated excellent clinical outcomes with minimal complications (3.3% bile leak rate, no major adverse events), attributable to three key technical optimizations. First, the combination of reverse Trendelenburg positioning with left lateral tilt and strategically placed high subxiphoid ports enabled gravity-assisted displacement of the transverse colon and omentum, while allowing instrument-leveraged liver retraction for optimal Calot’s triangle exposure. Second, our strict adherence to the “cold-cutting” principle^7^ was critical-using electrocautery in cut mode exclusively (< 45W) until initial bile flow visualization, followed by meticulous blunt dilation of the choledochotomy site, significantly reduced thermal injury risks. Third, Continuous biliary closure using 3-0 polydioxanone (PDO) barbed absorbable suture with meticulously maintained 1.0–1.5 mm stitch intervals achieved an exemplary postoperative bile leakage rate of 3.3%—a statistically and clinically significant improvement over the 7.1–12.5% range reported in contemporary literature^3,4^. Of particular clinical importance, preoperative MRCP evaluation proved essential for patient, as narrow bile ducts (≤ 1 cm diameter) demonstrated higher complication rates, with literature reporting 18.4% 5-year stricture risk in sub-8 mm ducts undergoing primary closure^3,7^.

The triple-port laparoscopic approach combining cholecystectomy and T-tube-free choledochoscopic common bile duct exploration demonstrates favorable short-term outcomes in patients with acute abdominal pain secondary to CBDS and cholecystolithiasis with acute cholecystitis, establishing its clinical safety and feasibility for broader adoption. However, this study’s limitations—particularly the modest sample size (n = 61) and restricted 3-month follow-up period—necessitate validation through multicenter RCTs to assess long-term efficacy and rare complication risks.

## Data Availability

All data generated during this study are included in this published article. Further minor datasets are available from the corresponding author on reasonable request.

## Abbreviations

LCBDE: Laparoscopic common bile duct exploration
CBDS: Common bile duct stones
LC: Laparoscopic cholecystectomy
ERAS: Enhanced recovery after surgery
CBD: Common bile duct
RCT: Randomized controlled trial
ASA: American Society of Anesthesiologists
BMI: Body Mass Index
CVS: Critical View of Safety
MRCP: Magnetic Resonance Cholangiopancreatography
POD: Postoperative day
EST: Endoscopic sphincterotomy
ERCP: Endoscopic Retrograde Cholangiopancreatography
